# Geohazard Identification in Underground Mines: A Mobile App

**DOI:** 10.3390/s24248052

**Published:** 2024-12-17

**Authors:** Pedro Lopez, Nathalie Risso, Angelina Anani, Moe Momayez

**Affiliations:** Department of Mining and Geological Engineering, University of Arizona, Tucson, AZ 8572, USA; pedrolopez@arizona.edu (P.L.); angelinaanani@arizona.edu (A.A.); mmomayez@arizona.edu (M.M.)

**Keywords:** vision-based detection, deep learning, mine safety

## Abstract

Mining is a critical industry that provides essential minerals and resources for modern society. Despite its benefits, the industry is also recognized as one of the most dangerous occupations, with geotechnical hazards being a primary concern. This study introduces the hazard recognition in underground mines application (HUMApp), a mobile application developed to enhance safety within underground mines by efficiently identifying geotechnical hazards, specifically focusing on roof falls. Employing a convolutional neural network (CNN), HUMApp achieved an accuracy of 90%, with recall and F1-score also reaching 90%, reflecting its high reliability in hazard detection. The application’s effectiveness was validated against expert assessments, showing significant agreement in identifying critical hazards. This validation highlights HUMApp’s potential to enhance proactive risk management in underground mining. This paper details each step of HUMApp’s development, from dataset preparation and model training to performance evaluation and application design, showcasing a scalable solution adaptable to various mining environments.

## 1. Introduction

Mining is an essential industry that plays a vital role in our daily lives, as it provides a wide range of critical resources and minerals, which are essential for modern technology, as well as for enabling the transition to low-carbon energy sources. Mineral resource extraction can be carried out through various methods, including underground mining, open-pit mining, and on water-based sources, depending on the location and type of the resource being extracted. Despite its many forms, all types of mining have inherent risks. Underground mining is often considered one of the most demanding and hazardous environments for workers, as they can be exposed to toxic emissions, dust, heat stress, and many hazards associated with cave-in and rockfalls. Government agencies like the Occupational Safety and Health Administration (OSHA) and the Mine Safety and Health Administration (MSHA) work hand in hand with mining companies to ensure that rigorous safety standards are met. For this purpose, strict construction and mining standards are in place and regular inspections are conducted in underground mines to proactively identify and mitigate any potential hazards. These inspections, however, strongly rely on workers’ ability to identify hazards, and are thus prone to human errors. In addition, hazard recognition using visual inspection requires extensive training and may be challenging given the extension of mine sites and illumination challenges existing in many underground environments. One alternative to automate hazard recognition is the use of computer vision-based solutions. Several applications in mining environments commonly take advantage of computer vision systems, deployed through surveillance cameras, mobile equipment, or smartphone apps, to help identify hazards and keep work environments safe. Recent advances in machine learning and deep learning have contributed to these results, as increased progress has been made towards solutions that operate in industrial environments with more or less controlled illumination. In underground mining, however, the incorporation of these technologies has faced slower advances, due mostly to the challenging conditions in underground mines, due to environmental variability as the operation progresses, challenging illumination, and a severe lack of representative data associated with structural hazards in particular. In spite of these challenges, the integration of advanced technologies such as computer vision and deep learning has increased its promise in enhancing traditional inspection practices. The incorporation of deep learning allows computer vision systems’ robustness to be improved, thus contributing to increased interest in the study of hazard recognition in more challenging environments, such as those found in underground tunnels and mines. Recently, several studies assessing tools for underground mine safety applications have been published, thus emphasizing structural hazard recognition as a growing field. In [1], the researchers evaluated the performance of machine learning (ML) and deep learning models (DL) in classifying seismic events in mining environments, showing that several commonly used models can achieve at least 91% accuracy, with the deep convolutional neural network (DCNN) model achieving a 94% overall accuracy. The work in [2] used convolutional neural networks (CNNs) to identify the time delay of arrival (TDOA) and location of micro-seismic events in underground mines, reducing the TDOA error from 3.29 ms to 1.16 ms. Other relevant studies include [3], who presented a deep learning-based approach for the automated identification of fissure traces in the mining industry, successfully segmenting various fissures and achieving accuracy comparable to human annotation; Ref. [4] also investigated the use of computer vision to detect adverse geological discontinuities and can provide detailed maps, aiding geotechnical analysis techniques. Another study related to the use of new technologies on geotechnical hazards in underground mines is presented in [5]. Here, researchers proposed digital image processing based on edge detection and showed that this methodology can be used to reduce the noise caused by non-uniform illumination and mutual interference of multi-scale edges, to identify cross-joint traces.

Another commonly researched hazard in this field is the risk of roof falls. According to MSHA, roof falls are defined as rock, earth, or debris falling from the roof or the walls in an underground operation. They are responsible for a significant number of accidents that can lead to fatalities, injuries, and damage to equipment. The identification of roof falls has caught the attention of many research teams, including [6,7], where computer vision and deep learning models, notably CNNs, were employed to identify roof falls in underground mines. The accuracy rates achieved were 89% and 86.4%, respectively, and both studies considered the problem of distinguishing between two categories for the classification. Similarly, Ref. [8] proposes the use of the CNN YOLOv7 (You Only Look Once) to identify bolts and meshes that could prevent roof falls in a coal mine roadway, achieving an accuracy of 95.4%, also considering two categories for the classification. Overall, these studies demonstrate the potential of computer vision and deep learning techniques in identifying geotechnical hazards in underground mines.

Building on the promise of deep learning for hazard identification, this work is focused on the development of a methodology for geotechnical hazard identification, specifically roof falls, in underground mines using deep learning models. The contributions of this work include, in particular, (1) a representative dataset generation for roof fall hazards, using a systematic methodology based on MSHA recommendations, suitable to be replicated in other underground environments; (2) the development and validation by safety experts of a deep learning-based model to assess roof fall hazards under a multi-class approach, often not found in the existing literature, which focuses on binary classification; and (3) the adaptation of the deep learning model into a computer app, by considering a reduced-parameter model, suitable to be used in smart devices, that can aid non-expert users in the identification and mapping of roof fall hazards and mine areas which require maintenance. The methodology and the app developed in this work are intended to be integrated with current inspection practices employed by miners and to provide a tool for more continuous hazard monitoring and management in underground environments. To assess the latter, the app performance was validated in a real underground mining environment at the University of Arizona’s San Xavier mining laboratory, located south of Tucson, AZ, USA.

## 2. Methodology

**Figure 1:**
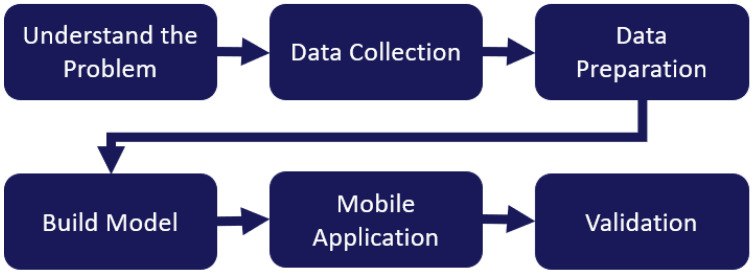
Stages of application development.

This section provides an overview of the methods considered in the development of this work. We start by providing a problem statement and describing how it relates to the current practices for hazard identification. Proper definitions, procedures, and visual cues considered for geotechnical hazard inspection are provided, as they serve as guidelines for identifying many of the problem requirements and relevant metrics to be considered. Data collection, filtering, preprocessing, and model development are described next. Finally, we discuss the development of the mobile application to ensure it meets user needs and industry standards. The stages used to develop this application are shown in Figure 1.

### 2.1. Problem Statement

According to the MSHA and their Roof Control Plan Approval And Review Procedures [9], each mine operator is required to develop and follow a roof control plan approved by the MSHA District Manager. This plan must be tailored to the prevailing geological conditions and the mining system used at the mine, including details for managing the roof, face, ribs, and addressing potential rock bursts. The plan must provide essential information to ensure supervisors and miners can maintain effective roof control and must be formally written and approved prior to implementation. Regular and thorough inspections are mandated to ascertain that roof control plans are comprehensive and comply with safety regulations. If a plan is found lacking, it is not approved until all deficiencies are rectified. The MSHA mandates that roof control plans be reviewed twice a year to verify ongoing effectiveness and to make adjustments in response to changes in mining conditions. There are specific metrics used in this evaluation process, including roof fall incident rates, injury rate related to roof falls, the frequency and severity of rock bursts, and the number of citations issued. These metrics are crucial for assessing the roof control plan effectiveness (for additional information, see MSHA Roof Control Plan Approval and Review Procedures). Regular inspections of roof control plans are critical for ensuring adherence to these safety standards. These inspections are typically conducted periodically by a senior geotechnical specialist, but every miner (or worker) is also responsible for examining their work area and reporting any potential hazards. Key aspects evaluated during these inspections include the following.

- Correct installation of ground support elements: Inspectors check for appropriate bolt spacing, ensuring that bolts are evenly distributed and securely anchored. This also includes verifying that mesh overlap is sufficient to cover and support the rock surface and that shotcrete (a type of sprayed concrete) is applied at the correct thickness to provide adequate support and prevent rockfalls [10].
- Condition of ground support elements: This involves assessing the physical state of support structures. Inspectors look for signs of spalling (chipping or flaking of the shotcrete), any damaged shotcrete that may compromise structural integrity, and the condition of bolts and mesh, to ensure they are intact and not deformed or broken.
- Presence of corrosion or corrosive conditions: Corrosion can weaken metal support elements, such as bolts and mesh. Inspectors look for rust or other signs of corrosion and assess the surrounding environment for conditions that could accelerate corrosive processes, such as high humidity or exposure to water.
- Changes in rock mass quality: The quality of the rock mass is crucial for mine stability. Inspectors examine joint characteristics (e.g., spacing, orientation, and infill material), the extent and nature of fracturing, and types of alteration (chemical changes in the rock) to determine any changes that could affect stability.
- Lithological or geotechnical-domain alterations: Changes in the geological composition or properties of rock layers (lithology) can affect the stability of the mine. Inspectors monitor for alterations in geotechnical domains (zones with distinct geological features) that could indicate potential instability or require changes in support strategies.
- Changes in water conditions or inflows: Water can significantly impact mine stability. Inspectors check for new or increased water inflows, changes in groundwater levels, and the presence of leaks, which can weaken rock and soil or lead to erosion and washout of support materials.
- Signs of structural movement or stress: Indicators of structural movement include cracking of shotcrete, deformation of support elements, and floor heave (upward movement of the mine floor). These signs suggest that the ground is shifting, which could lead to instability or collapse if not addressed.
- Presence of major geological structures: Faults, folds, and other major geological structures can create zones of weakness. Inspectors identify and monitor these structures to assess their potential impact on mine stability and plan appropriate support measures.
- Orientation of structures that could lead to wedge failure: Certain orientations of joints, faults, or bedding planes can create wedge-shaped blocks of rock that are prone to slipping or falling. Inspectors analyze the geometry and orientation of these structures to anticipate and prevent wedge failures.
- Evidence of mining irregularities: Over-break (excessive excavation beyond the planned boundaries) and poor scaling (failure to remove loose rock from walls and roofs) can compromise mine stability. Inspectors check for these irregularities to ensure that the mine is excavated and maintained according to plan.
- Visible tension cracks, raveling behind shotcrete, and other signs of instability: Inspectors look for surface cracks, especially those indicating tension, which suggest that the rock is under stress and may fail. Raveling (falling of small rock pieces behind shotcrete) and other signs of instability are also closely monitored to prevent larger rockfalls or collapses.

Although it is the responsibility of every miner to identify and report any of these characteristics, the reality is that most miners may not have the expertise to report this effectively. A portable tool that can aid workers in identifying hazards can be a valuable tool to improve safety conditions in underground mines, and potentially also in other underground environments, such as tunnels, subway infrastructure after an emergency, etc. Therefore, this paper proposes the development of a mobile application, HUMApp, designed to assist miners in identifying and reporting potential geotechnical hazards related to roof falls. While inspectors evaluate a comprehensive range of variables, it is not feasible for our application to identify all those variables. Instead, HUMApp focuses specifically on helping miners recognize visual clues indicative of such hazards, such as visible cracks, corrosion, protruding bolts, and other structural irregularities. By providing an easy-to-use interface, HUMApp aims to support regular inspections and enhance compliance with safety regulations. This will enable every miner to identify preliminary possible hazards and promptly inform experts for a more in-depth inspection, thus contributing to reducing the occurrence of roof fall accidents in underground operations.

#### Requirements

In order to provide a tool suitable to contribute to hazard identification according to the guidelines described in the previous section, we have defined, in consultation with mine operators, the following requirements to ensure functionality in an underground environment:

- Accuracy and type II errors (false negatives): The application must accurately identify and categorize geotechnical hazards, particularly those related to roof falls. In particular, it is required to obtain not only an acceptable accuracy, but also to reduce the number of false negatives, as these can lead to identifying hazards as non-hazards, thus leading to safety risks.
- Robustness: The application must perform reliably in conditions typical of underground environments, including areas with low visibility and high dust levels.
- Ease of installation and use: The application must be simple to install and user-friendly for all miners.
- Repeatability: The development methods should be replicable and adaptable for use in other mines or underground environments subject of similar hazards.

By meeting these requirements, we aim to develop an application that enhances safety practices and supports miners in their daily operations. To ensure that these requirements are adequately addressed, a survey was designed to evaluate the application’s ease of use and installation, as well as to assess its overall performance in a typical underground environment. The survey specifically targeted end-users to gather feedback on the application’s reliability and functionality under real-world conditions. Additionally, the accuracy of the application was validated by experts in the field, who provided insights into its reliability in identifying and categorizing geotechnical hazards.

### 2.2. Experimental Design

Additionally to the MSHA Roof Control Plan Approval and Review Procedures described in Section 5, we consulted with three senior geotechnical engineers from various mining operations and consulting firms. Although not exhaustive, these consultations helped us define the most important visual aspects to evaluate during underground mining inspections for roof fall identification. With this information, we established four primary categories for classification within the application: Hazard, Maintenance Required, Not Hazard, and Not Usable. A summary of these guidelines is presented in Table 1, which outlines the criteria for each category.

Following the categorization framework, the next step was to design the methodology for model development. Characterizing hazards in underground environments is a critical task; however, unlike the case of other application domains for computer vision, such as “classic” object recognition, few public data currently exists for the development and refinement of specialized machine learning models. In addition to this challenge, the labeling of such data represents a labor-intensive and time-consuming task, which needs to be validated by personnel with the proper expertise. For the model development presented here, we generated a dataset consisting of images captured in a real-life underground mining environment, under the supervision of trained safety professionals. The main guidelines used for data collection are described next:

- Photographs should be taken directly in front of the target area to be inspected.
- The camera should be positioned one meter away from the target.
- The frame should be free of people and extraneous elements.
- Images must be clear and not blurred.

With these criteria set, we proceeded to collect the dataset necessary for training our deep learning model.

### 2.3. Dataset

The dataset used in this study was captured from The San Xavier (SX) Mining Laboratory, located approximately 37 km south of Tucson, Arizona. The SX laboratory consists of a real mine with 4 underground levels, reaching up to a depth of 330 ft; it contains silver, lead, zinc, and copper, characteristic of southern Arizona deposits. This laboratory, affiliated with the University of Arizona, comprises around 900 m of underground workings spanning three levels. The dataset consists of 2817 images captured within the underground mine, collected using a combination of Android-based and iOS-based mobile devices. The use of different cameras allowed the acquisition of images with diverse image quality, enhancing the robustness of our dataset.

- Camera 1: Samsung Galaxy A73 with a maximum resolution of 1080 × 2400 pixels (video), 108 MP primary camera with f/1.8 aperture, 24 mm equivalent focal length, optical image stabilization (OIS), and a diagonal field of view of 80° (visible spectrum).
- Camera 2: iPhone 14 Pro Max with a maximum resolution of 1920 × 1080 pixels (video), 48 MP primary camera with f/1.78 aperture, 24 mm equivalent focal length, second-generation sensor-shift optical image stabilization (OIS), seven-element lens, 100% focus pixel resolution, and a diagonal field of view of 78° (visible spectrum).

Following the completion of the dataset collection, each image was manually classified into one of the four categories previously outlined in Section 2.2. The classification process adhered strictly to the criteria detailed in Table 1.

**Figure 2:**
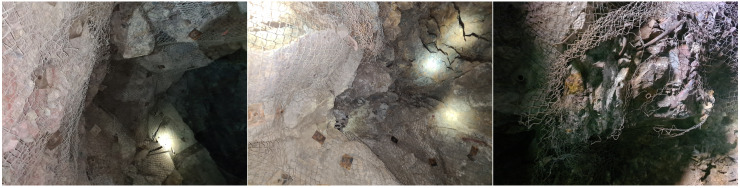
Example of the Hazard class—characterized by unsupported areas, and the presence of discontinuities.

**Figure 3:**
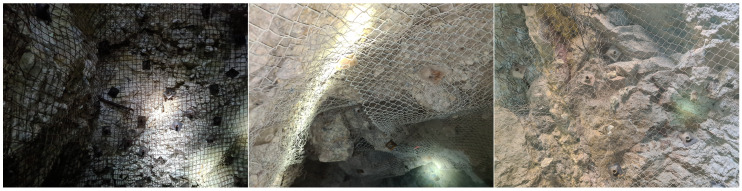
Example of the Maintenance Required class—presenting bulging mesh, indicative of necessary maintenance.

**Figure 4:**
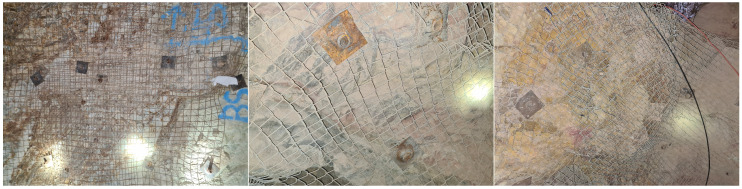
Example of the Not Hazard class—characterized by the absence of discontinuities and proper support provided by mesh and bolt.

**Figure 5:**
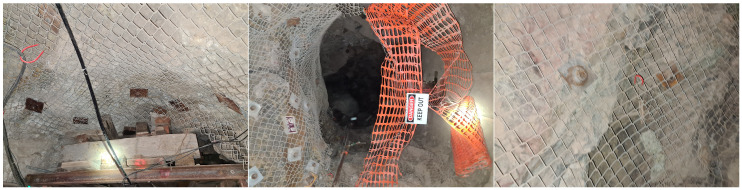
Example of the Not Usable class—displaying a moving image, rendering it unsuitable for hazard assessment.

To provide a visual understanding of each category, examples are shown in Figure 2, Figure 3, Figure 4 and Figure 5. The distribution of images after labeling was as follows: 1239 in the “Hazard” class, 421 in the “Maintenance Required” class, 713 in the “Not Hazard” class, and 444 in the “Not Usable” class.

To enhance our dataset and ensure class balance, we implemented a series of transformations on the original set of 2817 images. Despite following specific criteria and developing guidelines for capturing these images, variations inevitably arise due to factors such as differences in the heights of the individuals taking the photos, and the fact that pictures are not always taken from exactly the same distance or position. Although these variations affect data capture, they were chosen for the data collection procedures as they mimic the conditions in which a diverse workforce may capture images for the intended application in real-life scenarios. To simulate a broader range of conditions and improve the robustness of our deep learning model, we applied data augmentation techniques such as rotations, translations, flipping, and zooming. These transformations help mimic the diverse perspectives and distortions that occur in real mining environments, where conditions are not always ideal and can vary considerably. By expanding our dataset in this manner, we not only balanced the class distribution but also increased the total number of images to 10,000. While it is possible to use any number of images to enhance the dataset, 10,000 images were deemed sufficient to achieve our objectives without imposing excessive computational demands. This number ensures that the model is both effective and efficient, providing reliable hazard detection in underground mining operations. Additionally, this expansion allows for an equal distribution of 2500 images in each class, enhancing the model’s exposure to varied data and thereby improving its predictive accuracy and reliability.

Furthermore, all images in our dataset were resized to a standardized dimension of 224 × 224 pixels. This uniformity is crucial as it streamlines the input process, ensuring that our deep learning application can efficiently handle the data during training. Additionally, we applied scaling and normalization techniques to the images. This process involves re-scaling the channel values of each image to a range between 0 and 1, which is a standard practice in image processing for neural networks. Such scaling and normalization help in reducing model training times and improve the convergence behavior during the learning process. By standardizing the pixel intensity values across all images, we also mitigate issues related to variations in lighting and exposure that can occur in different mining environments. This rigorous preprocessing ensures that our dataset is well prepared, making it more conducive for the deep learning model to learn meaningful patterns and features effectively and accurately.

### 2.4. Model Development

Once data collection and augmentation was completed, deep learning-based model development was performed. Deep learning, a subset of machine learning, leverages algorithms inspired by the human brain’s structure and function, known as artificial neural networks. This approach is distinct from traditional machine learning techniques due to its use of multiple layers that can model complex patterns and relationships within large datasets. While some machine learning models also utilize multiple layers, the fundamental difference lies in the depth and complexity: machine learning models generally consist of a few layers, whereas deep learning models are distinguished by their many layers, sometimes hundreds. Another crucial difference is that traditional machine learning models typically require manual feature extraction, where specific characteristics or features are identified and fed into the model. In contrast, deep learning models have the ability to automatically learn and extract relevant features from raw data during the training process. This deep architecture and feature-learning capability allow deep learning to excel at recognizing complex patterns in unstructured data, such as images [11].

Convolutional neural networks (CNNs), a specialized class of deep neural networks, are tailored for processing structured grid data like images. CNNs employ learnable filters to perform convolution operations on input data, making them highly effective for tasks such as image classification, object detection, and segmentation. These tasks require precise identification and spatial distribution of objects within images [12]. Based on our literature review, CNNs are the preferred model for studying geotechnical hazards in underground mines using deep learning techniques due to their ability to handle complex visual data [13].

In our project, the primary role of the CNN is to classify each image into one of four predefined categories, presenting a clear classification challenge. The images, once preprocessed and standardized, are fed into the CNN. The network then utilizes its learned filters to detect and categorize key features that indicate potential hazards or maintenance requirements in the mining environment.

To determine the most effective CNN architecture for our application, we reviewed and compared various alternatives commonly used in the literature, selecting the most suitable based on performance. To assess the performance of these models, we employed several key metrics.

#### 2.4.1. Metrics

We utilize several metrics to evaluate different aspects of the model’s predictive abilities, including accuracy, recall, and F1−score. Additionally, considering that this application is being developed for a mobile device, it is essential to account for other factors such as model complexity and execution speed. Therefore, we also include metrics such as model size (number of parameters) and computational efficiency in giga floating point operations per second (GFLOPs) [14].

- Accuracy
  This is one of the most intuitive performance measures. It represents the ratio of correctly predicted instances to the total instances in the dataset. The equation for accuracy is given by
  (1) $$\mathrm{Accuracy}=\frac{True\;Positives+True\;Negatives}{Total\;Instances}$$
  where
  Accuracy: The proportion of true results among the total number of cases examined;
  Truepositives: The correctly predicted positive instances;
  Truenegatives: The correctly predicted negative instances;
  Totalinstances: The total number of instances examined.
- Recall
  Also known as sensitivity or true positive rate, recall measures the proportion of actual positives that are correctly identified. It is particularly important in scenarios where the cost of false negatives is high. The recall is calculated as
  (2) $$\mathrm{Recall}=\frac{True\;Positives}{True\;Positives+False\;Negatives}$$
  where
  Recall: The proportion of actual positives that were correctly identified;
  Truepositives: The correctly predicted positive instances;
  Falsenegatives: The positive instances that the model incorrectly predicted as negative.
- F1−Score
  The F1−score is the harmonic mean of precision and recall. It is a more reliable measure than accuracy, especially in cases of imbalanced datasets. The equation for the F1−score is
  (3) $$F1=2\times \frac{Precision\times Recall}{Precision+Recall}$$
  where
  F1: The harmonic mean of precision and recall, providing a balance between them;
  Precision: The ratio of truepositives to the sum of truepositives and falsepositives;
  Recall: The proportion of actual positives that were correctly identified;
  Truepositives: The correctly predicted positive instances;
  Falsepositives: The negative instances that the model incorrectly predicted as positive.
  In the context of our application, where safety is crucial, there is some metrics that are more relevant. Our approach prioritizes recall, ensuring that as many hazards as possible are identified, even if this means occasionally classifying a non-hazard as a hazard (falsepositive). This approach is safer and more effective in an underground mining environment, where the consequences of overlooking a hazard could be severe.
-
ModelComplexity

  Modelcomplexity is assessed by the number of parameters in the model. It provides an indication of the model’s size and potential computational demands.
  (4) ModelComplexity=Totalnumberofparameters
  where
  Modelcomplexity: The total number of learnable parameters in the model.
- Execution Speed
  Execution speed is measured by the number of GFLOPs: required for a single forward pass. It indicates the computational efficiency of the model.
  (5) $$GFLOPs=\frac{\mathrm{Number}\;\mathrm{of}\;\mathrm{floating}\;\mathrm{point}\;\mathrm{operations}}{10^{9}}$$
  where
  GFLOPs: The number of floating point operations in billions required for a single forward pass through the model.

Each metric provides a unique perspective on the strengths and weaknesses of a model, enabling a thorough evaluation. By assessing accuracy, recall, and F1-score, we gain insights into the overall effectiveness of a model, i.e., its ability to capture all relevant instances.

As mentioned, to identify the most suitable CNN architecture for our application, we conducted a comprehensive comparative analysis of several CNN architectures. These included MobileNet, MobileNetV2, ResNet-50, InceptionV3, VGG16, DenseNet, and EfficientNet. Each model was selected based on our literature review, which highlighted their effectiveness in similar applications and their ability to handle complex image data efficiently.

The performance results of these CNN architectures are summarized in Table 2. This comparison highlights MobileNetV2 and DenseNet as the standout performers, exhibiting the most promising results in terms of accuracy, recall, and F1-score. MobileNetV2 achieved an F1-score of 90%, close to DenseNet, while significantly outperforming other architectures like MobileNetV3 and ResNet-50, which yielded F1-scores of 53% and 55%, respectively. These results demonstrate MobileNetV2’s ability to achieve high accuracy while maintaining a compact architecture suitable for mobile applications.

MobileNetV2’s superior performance can be attributed to its unique architectural features, such as depthwise separable convolutions, linear bottlenecks, and inverted residuals. These features enable efficient feature extraction and reduced computational complexity, making it particularly well suited for real-time applications in mobile environments. DenseNet, while achieving similar accuracy metrics, is less computationally efficient, limiting its practical application in mobile scenarios. Conversely, models like MobileNetV3 and ResNet-50, despite being widely regarded as efficient architectures, did not perform as well on this specific dataset. EfficientNet’s low F1-score highlights its unsuitability for this specific application, possibly due to overfitting or inefficiencies in processing this dataset. InceptionV3 and VGG16 performed relatively well but fell short of the top-performing models, suggesting their architectures are not the best fit for this specific task compared to MobileNetV2 and DenseNet, as has also been documented in other studies [15,16].

Given the specific requirements of our project to develop a mobile application, we chose the MobileNetV2 architecture for our model. This decision was based on MobileNetV2’s exceptional balance of efficiency and performance, which is crucial for the real-time processing needed in mobile environments.

#### 2.4.2. MobileNet

MobileNetV2 is an advanced neural network architecture designed primarily for mobile and embedded vision applications, famous for their lightweight deep learning models that optimize speed and efficiency. MobileNetV2 introduces several innovative architectural features that significantly enhance its performance [17]:

- Depthwise separable convolutions: These are used to reduce the model size and computational cost by separating the convolution into a depthwise and a pointwise convolution, effectively filtering inputs and combining them to create new features.
- Linear bottlenecks: These structures capture the important features of the network and compress the input information to reduce dimensions, which conserves processing power without significant loss of information.
- Efficient model architecture: MobileNetV2 optimizes both the architectural design and the operation flow to maximize efficiency. This enables the model to deliver high accuracy while maintaining a small footprint, making it ideal for devices with limited computational resources.

The architecture of MobileNetV2, as illustrated in Table 3, has a design that balances high performance with low computational demands, making it ideal for resource-constrained environments like mobile devices.

The structure of MobileNetV2 not only decreases the computational burden but also maintains high accuracy, making it highly effective for a variety of real-world applications such as image classification. It is particularly well suited for scenarios where computational resources are limited, such as smartphones, IoT devices, and other embedded systems [18].

MobileNetV2 has demonstrated its capability to achieve comparable accuracy to more computationally intensive models while being substantially faster and lighter. This combination of speed, efficiency, and accuracy sets MobileNetV2 apart as a leading architecture for mobile vision applications and an ideal choice for our project, providing a powerful tool for deploying high-performance AI models on resource-constrained devices.

To adapt MobileNetV2 for geotechnical hazard detection, we applied transfer learning by freezing the pre-trained feature extraction layers and replacing the final dense layers to suit our classification task. The architecture modifications are outlined in Table 4.

**Figure 6:**
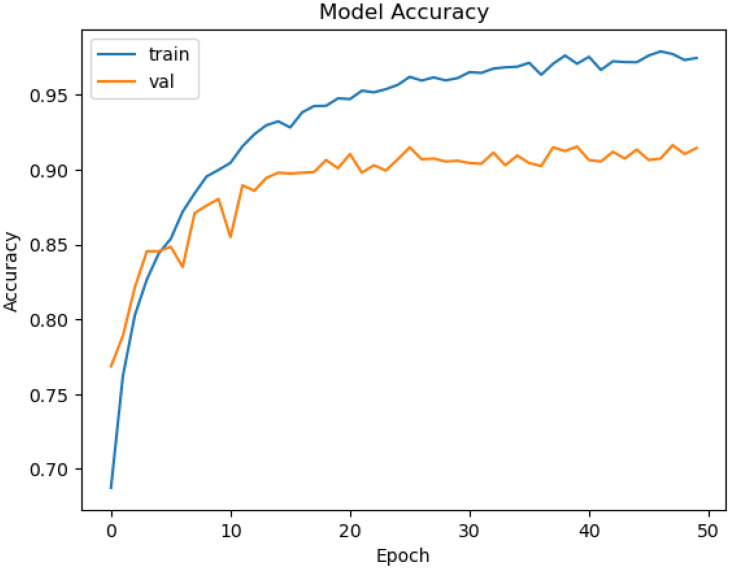
Training and validation accuracy for MobileNetV2.

The model consists of 2,915,908 parameters, of which 657,924 are trainable, reflecting the fine-tuning of dense layers while leveraging the pre-trained MobileNetV2 base. Figure 6 illustrates the training and validation accuracy curves; each epoch required approximately 24 s, and the total training process completed within 15 min. The model achieved a training accuracy of approximately 95% and a validation accuracy of 90%.

Table 5 summarizes the model’s classification performance. It achieved an overall accuracy of 90%, with precision, recall, and F1-scores consistently at 90%. The class-wise metrics reveal slight variability, with precision ranging from 86% to 94%.

An analysis of the misclassifications highlights several contributing factors, such as ambiguous visual features that may confuse the model. For instance, conditions like low luminosity or image blurriness were often categorized as ’Not Usable’ by the application, which aligns with the inherently subjective nature of such assessments. These findings suggest areas for improvement, particularly in enhancing the model’s sensitivity and adaptability to challenging visual conditions, ensuring more accurate classifications even under suboptimal circumstances.

### 2.5. Mobile Application

Following the completion of our deep learning model, we proceeded with the development of the mobile application. We chose Flutter, a versatile UI framework by Google, for designing a user-friendly interface for our mobile application. To integrate our deep learning model into the mobile environment, we used TensorFlow Lite (TFLite), a framework designed for efficient machine learning on mobile devices. This section describes the process of embedding the TFLite model into a Flutter application, providing insights into the overall app design.

#### 2.5.1. TensorFlow Lite

TensorFlow Lite is an open-source deep learning framework developed by Google, designed specifically for on-device inference with low latency and a small binary size. TensorFlow Lite enables the deployment of machine learning models on mobile and embedded devices, supporting platforms like Android and iOS, as well as IoT devices. While most of the platforms are utilized in different commercial applications, in the literature, we observe that the most popular toolkit is TensorFlow Lite [19,20,21].

#### 2.5.2. Flutter

Flutter is an open-source UI software development kit created by Google. It is used for developing cross-platform applications for Android, iOS, Linux, Mac, Windows, Google Fuchsia, and the web from a single codebase. Flutter’s key features include the following [22]:

- Fast development: Flutter’s Hot Reload allows developers to instantly see changes in the code, enabling quick experimentation, UI building, feature addition, and bug fixing.
- Expressive and flexible UI: Flutter enables the creation of high-quality, natively compiled applications for mobile, web, and desktop from a single codebase.
- Native performance: Flutter’s widgets incorporate all critical platform differences such as scrolling, navigation, icons, and fonts to provide full native performance on both iOS and Android.

**Figure 7:**
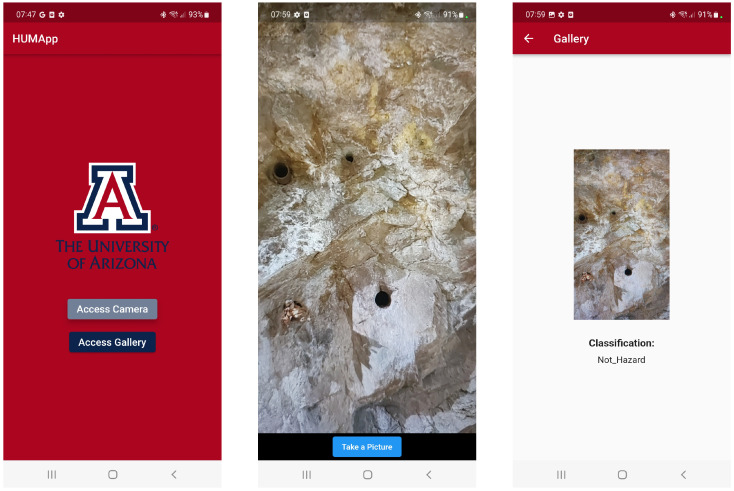
Screenshots of the mobile application: (**Left**) App homepage, allowing access to the camera or gallery. (**Center**) Camera view displaying. (**Right**) Classification result showing the identified category for the image.

**Figure 8:**
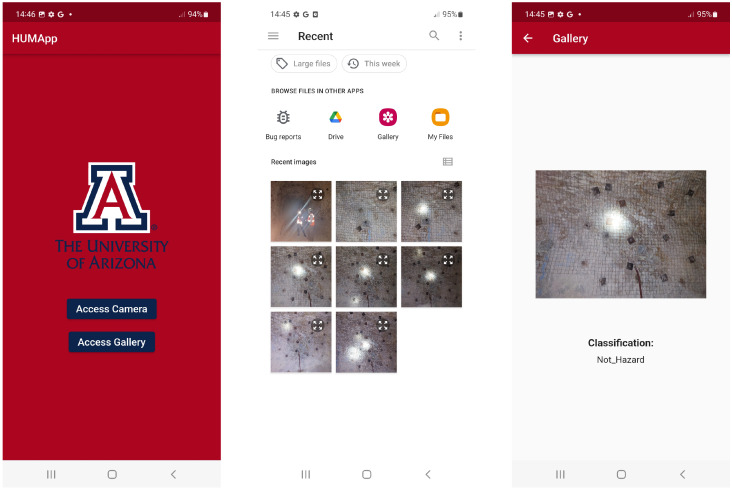
Screenshots of the mobile application: (**Left**) App homepage, allowing access to the camera or gallery. (**Center**) Gallery view, displaying stored images for processing. (**Right**) Classification result, showing the identified category for an image.

Following the conversion of our deep learning model to TensorFlow Lite, we integrated this streamlined version into our Flutter-based mobile application. The final version of the application’s user interface across different mobile devices is illustrated in Figure 7 and Figure 8. The interface features a user-friendly design with two prominent buttons for capturing or uploading a photo from a smartphone and subsequently classifying the image automatically.

## 3. Results

**Figure 9:**
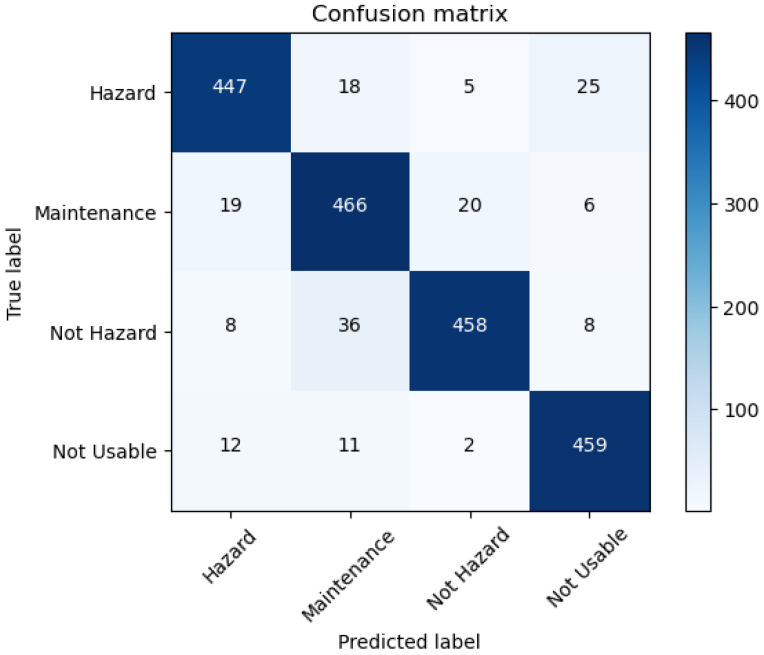
Confusion matrix.

Finally, in this section we present the key findings and outcomes of the project, showcasing the effectiveness of the developed application. The performance of the application is visually depicted in the confusion matrix shown in Figure 9. From this matrix, key metrics such as accuracy, recall, and F1-score were calculated.

- Accuracy: The application achieved an overall accuracy of 90%, indicating a high rate of correct predictions across all categories. This metric reflects the proportion of total correct predictions out of all predictions made, demonstrating the model’s reliability in identifying geotechnical hazards.
- RecallandF1-score: With a score of 90% in both recall and F1-score, the model effectively minimizes missed hazard detections, a critical aspect for safety applications.
- Model size: The model consists of 2,915,908 parameters. This relatively small number of parameters contributes to the model’s efficiency and makes it suitable for deployment on mobile devices.
- GFLOPs: The model requires 0.614 GFLOPs (giga floating point operations) per forward pass. This low computational requirement ensures that the model can perform real-time hazard detection on resource-constrained devices, such as mobile phones.

### App Validation

**Figure 10:**
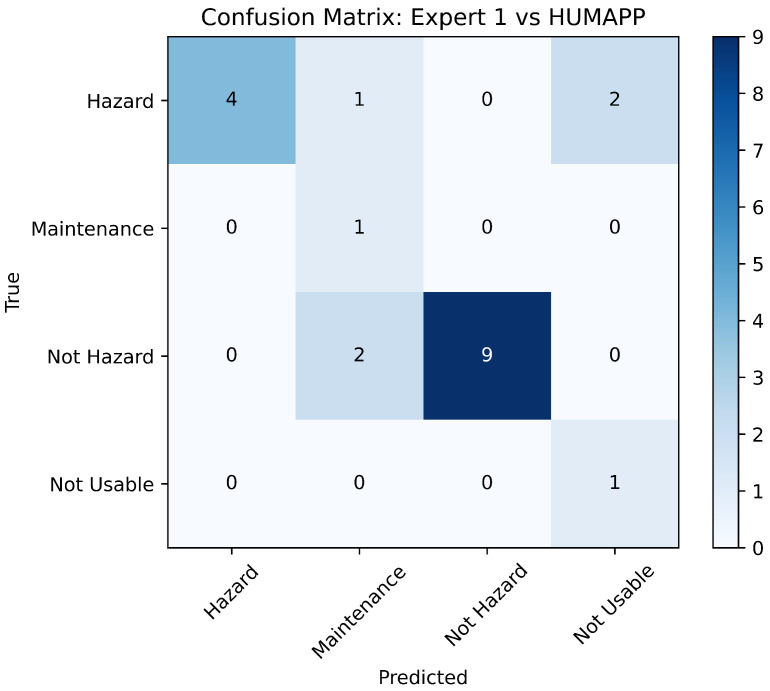
Comparison of HUMApp versus expert 1.

**Figure 11:**
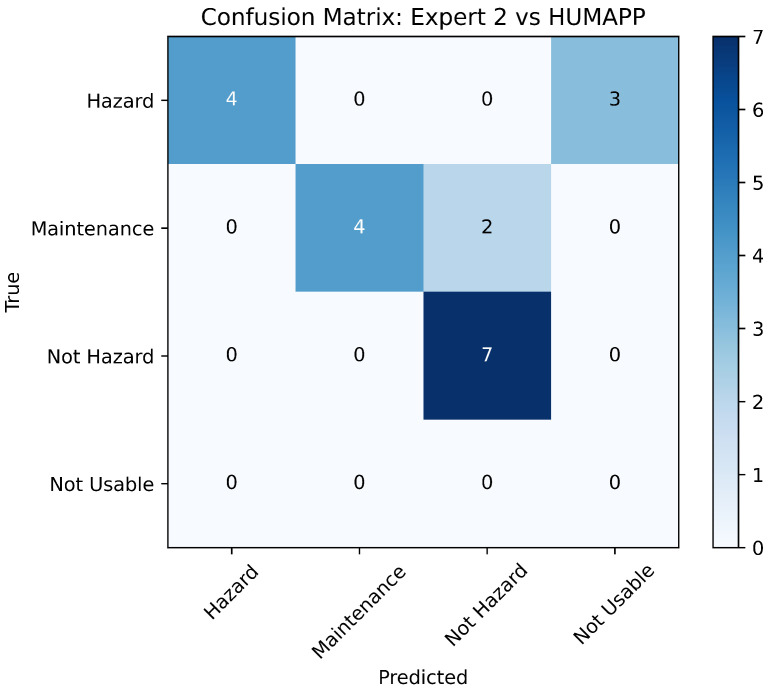
Comparison of HUMApp versus expert 2.

To validate the effectiveness of our application, we engaged in a comparative analysis with assessments from two field experts, using a validation dataset of 20 new images. This approach aligns with our requirements to ensure the app’s accuracy through expert benchmarking. Each expert independently assessed the dataset, and their classifications were compared to those made by our application. The comparative results, shown in Figure 10 and Figure 11, highlight the application’s alignment with expert judgments in essential safety categories.

The analyses indicate significant concordance between the app’s classifications and the experts, particularly in pivotal categories such as ‘Hazard’ and ‘Not Hazard’. This demonstrates the app’s precision in identifying potential risks with a high degree of reliability, which is essential for fulfilling the safety-critical requirements of mining operations.

However, some discrepancies were noted in the ‘Maintenance Required’ and ‘Not Usable’ categories, likely due to the inherently subjective nature of these assessments. Experts might perceive conditions such as low luminosity or blurriness that the app categorizes as ‘Not Usable’, pointing to areas for further enhancement in terms of app sensitivity and adaptability.

Despite these variations, the overall agreement with expert opinions strongly supports the robustness and consistency of HUMApp’s approach to image categorization. This validation not only confirms the application’s efficacy but also its alignment with the initial project requirements for creating a reliable and user-friendly tool for hazard identification in underground mining environments.

## 4. Discussion

In this section, we discuss the implications and limitations of our project, focusing on the methodology rather than the specific application developed. Our main goal was not to create a one-size-fits-all solution for all mining operations. Instead, we aimed to develop a flexible and adaptable methodology that can be replicated in different mining contexts. This approach ensures that the technology can be applied effectively across various geological and operational environments.

The development of the mobile application using the MobileNetV2 architecture and TensorFlow Lite within the Flutter framework exemplifies an effective approach to tackling geotechnical hazard identification in mines. However, it is crucial to recognize that each mining operation has unique characteristics and challenges. These include varying geological conditions, different scales of operation, and diverse regulatory environments. By presenting a flexible and scalable methodology, this project lays the groundwork for other mining operations to adapt and implement similar technologies tailored to their specific needs. The application’s architecture allows for modifications and tuning to address the particularities of different mining sites. This adaptability is crucial for the technology’s adoption in a sector as varied as mining. This project encourages further research and development in the area of machine learning applications in mining, especially in geotechnical hazard identification. By sharing our approach and findings, we aim to contribute to the ongoing discourse in the mining community about leveraging technology to enhance safety and efficiency. The successful replication of this methodology in other mines could lead to significant advancements in hazard detection and risk management, as well as contributing to the creation of specialized architectures for geotechnical hazard identification. Furthermore, the impacts of this research can be expanded to other types of underground infrastructures, such as tunnels, subway, construction environments, and other types of critical and transportation infrastructure that requires geotechnical hazard monitoring. In many of these cases, although some 3D sensing technologies exists for assessment, these are usually expensive and they do not necessarily allow for continuous monitoring that can be tuned for specific geological conditions.

## 5. Conclusions and Future Work

The development and validation of HUMApp represents a step forward in the use of deep learning models to improve underground mine safety protocols. This research achieves our goal of developing methods for a computer vision application based on a deep learning model capable of detecting preliminary roof falls threats. HUMApp, which was implemented at the San Xavier mining laboratory, Arizona, has proven to be extremely helpful in detecting key geotechnical hazards and thereby improving proactive risk assessment.

Moving forward, we plan to focus on refining HUMApp’s classification accuracy across different categories. By expanding the dataset and incorporating feedback from real-world deployments, we anticipate significant improvements in the model’s accuracy and adaptability to various mining conditions. This continuous development will not only enhance the model’s reliability but also its applicability to a broader range of mining operations.

Furthermore, we intend to augment HUMApp with georeferencing capabilities to not only detect hazards but also precisely map their locations within the underground mines. Integrating spatial mapping technology into the application will facilitate a more strategic approach to hazard management, allowing mine operators to prioritize interventions and optimize safety measures effectively.

## Figures and Tables

**Table 1:** Criteria for hazards.

Class	Criteria
Hazard	Areas where ground support is inadequate or failing, characterized by unsupported sections, visible discontinuities in the rock mass, protruding bolts, and bent plates. These signs indicate immediate danger, requiring prompt attention to prevent rockfalls or collapses.
Maintenance Required	Sections where ground support elements show signs of wear or minor failure, such as bulging mesh or bent plates. These areas are not immediately hazardous but need maintenance to prevent future deterioration into more severe conditions.
Not Hazard	Areas where the rock mass is fully supported, and with no visible discontinuities. Bolts and mesh are flush with the rock surface, indicating that the ground support is functioning as intended and there are no immediate risks.
Not Usable	Images that cannot be used for assessment due to obstructions, such as objects or people in the frame, or due to poor quality, such as blurry or moving images. These images need to be retaken to provide a clear view of the area being inspected.

**Table 2:** Comparison of model metrics.

CNN Architecture	Precision	Recall	F1-Score
MobileNetV2	90	90	90
MobileNetV3	54	53	53
ResNet-50	56	56	55
InceptionV3	88	88	88
VGG16	85	85	85
DenseNet	92	91	91
EfficientNet	6	25	10

**Table 3:** Summary of MobileNetV2 architecture.

Input	Operator	t	c	n	s
$224^{2}\times 3$	conv2d	-	32	1	2
$112^{2}\times 32$	bottleneck	-	16	1	1
$112^{2}\times 16$	bottleneck	6	24	2	2
$56^{2}\times 24$	bottleneck	6	32	3	2
$28^{2}\times 32$	bottleneck	6	64	4	2
$14^{2}\times 64$	bottleneck	6	96	3	1
$14^{2}\times 96$	bottleneck	6	160	3	2
$7^{2}\times 160$	bottleneck	6	320	1	1
$7^{2}\times 320$	conv2d 1x1	-	1280	1	-
$7^{2}\times 1280$	avgpool 7x7	-	-	-	-
1×1×1280	conv2d 1x1	-	k	-	-

**Table 4:** MobileNetV2 architecture details.

Layer (Type)	Output Shape	No. of Params
mobilenetv2_1.00_224 (Functional)	(None, 7, 7, 1280)	2,257,984
global_average_pooling2d (GlobalAveragePooling2D)	(None, 1280)	0
dense (Dense)	(None, 512)	655,872
dropout (Dropout)	(None, 512)	0
dense_1 (Dense)	(None, 4)	2052
Total Parameters		2,915,908
Trainable Parameters		657,924
Non-trainable Parameters		2,257,984

**Table 5:** Classification metrics for MobileNetV2.

Class	Precision	Recall	F1-Score
Hazard	0.89	0.92	0.90
Maintenance	0.84	0.89	0.86
Not Hazard	0.94	0.86	0.90
Not Usable	0.94	0.92	0.93

## Data Availability

The dataset employed in this study was exclusively captured at the San Xavier mine laboratory, operated by the University of Arizona, ensuring its origin is fully traceable and secure.
